# A Pediatric Case of Homocystinuria Due to MTHFR Deficiency with Early Clinical Features Resembling Leigh Encephalopathy

**DOI:** 10.3390/children13101332

**Published:** 2026-09-30

**Authors:** Tatsunori Itabashi, Jiro Abe, Midori Nakajima, Yuki Ueda, Kiyoshi Egawa, Masayoshi Nagao, Atsuhito Takeda

**Affiliations:** 1 Department of Pediatrics, Hokkaido University, Kita 15, Nishi 7, Sapporo 060-8638, Japan; itabashi.tatsunori.k0@elms.hokudai.ac.jp (T.I.); midori.nakajima@med.hokudai.ac.jp (M.N.); yu_ueda@med.hokudai.ac.jp (Y.U.); egakiyo@huhp.hokudai.ac.jp (K.E.); a-takeda@med.hokudai.ac.jp (A.T.); 2 Department of Pediatrics, Genetic and Metabolic Center, National Hospital Organization Hokkaido Medical Center, Sapporo 063-0005, Japan; nagao.masayoshi.nq@mail.hosp.go.jp

**Keywords:** homocystinuria due to MTHFR deficiency, methylenetetrahydrofolate reductase (MTHFR), Leigh encephalopathy, Leigh syndrome spectrum

## Abstract

**Highlights:**

**What are the main findings?**

• This patient had homocystinuria due to MTHFR deficiency with a phenotype resembling Leigh encephalopathy.
• The etiologies of Leigh encephalopathy are diverse, and mitochondrial dysfunction can occur secondary to metabolic disorders such as homocystinuria.

**What are the implications of the main findings?**

• The concept of the Leigh syndrome spectrum encompasses patients who fall within the disease spectrum of Leigh syndrome but present with atypical clinical features.
• Because the Leigh syndrome spectrum includes metabolic disorders, screening for metabolic abnormalities is essential to avoid missing potentially treatable diseases.

**Abstract:**

**Background**: Leigh encephalopathy is one of the most common phenotypes of mitochondrial diseases. It is characterized by progressive psychomotor regression, accompanied by elevated lactate/pyruvate ratios in blood and cerebrospinal fluid (CSF) and lactate on MRS, and symmetric lesions in the bilateral basal ganglia on brain MRI. A clinical diagnosis is made based on these findings and is confirmed by demonstrating mitochondrial dysfunction through genetic or enzymatic testing. However, some patients who present with clinical features of Leigh encephalopathy actually develop secondary mitochondrial dysfunction due to inborn errors of metabolism, thereby exhibiting a Leigh syndrome phenotype. **Case Presentation**: The patient was born at term without perinatal complications. Poor feeding was noted from two months of age, and microcephaly and hypotonia were identified at the 4-month health checkup. At 5 months, the patient was admitted for further evaluation. CSF analysis revealed an elevated lactate-to-pyruvate ratio of 21. Brain MRI demonstrated symmetrical T2-weighted hyperintensities in the bilateral basal ganglia, and MR spectroscopy detected a lactate peak in the same region. Based on clinical findings, CSF results, and MRI features, Leigh encephalopathy was suspected. At 9 months, mitochondrial DNA sequencing of skeletal muscle showed no significant mutations, and skeletal muscle biopsy revealed no findings suggestive of neuromuscular disease. At 7 years of age, two weeks after contracting COVID-19, the patient was admitted to the hospital with acute metabolic decompensation characterized by acidosis and complicated by a small intraventricular hemorrhage. Tandem mass spectrometry performed during the episode suggested homocystinuria due to MTHFR deficiency. Subsequent trio-based genetic testing revealed compound heterozygous variants in *MTHFR*. Measurement of MTHFR enzyme activity revealed a profound reduction, confirming the diagnosis of homocystinuria due to MTHFR deficiency. After initiation of specific metabolic therapy, including betaine, both urinary and plasma homocysteine levels decreased, and clinical symptoms improved. **Conclusions**: Homocystinuria due to MTHFR deficiency may cause secondary mitochondrial dysfunction, leading to a Leigh syndrome phenotype. When Leigh encephalopathy is suspected, metabolic screening should be performed to avoid missing potentially treatable diseases.

## 1. Introduction

Homocystinuria comprises a group of inherited disorders of homocysteine metabolism characterized by thromboembolic tendencies and neurocognitive manifestations resulting from hyperhomocysteinemia. The most common inherited defect of homocysteine metabolism is classical homocystinuria due to cystathionine β-synthase (CBS) deficiency. Another major metabolic route of homocysteine is the remethylation pathway, in which homocysteine is converted back to methionine via methionine synthase, a cobalamin-dependent enzyme that uses 5-methyltetrahydrofolate as a methyl-group donor. Homocystinurias due to impaired remethylation include homocystinuria due to disorders of intracellular cobalamin metabolism and homocystinuria due to methylenetetrahydrofolate reductase (MTHFR) deficiency. Whereas plasma methionine is typically elevated in classical homocystinuria due to CBS deficiency, it is usually normal or decreased in homocystinuria due to disorders of intracellular cobalamin metabolism and homocystinuria due to MTHFR deficiency, in which the remethylation pathway is impaired [1].

Leigh encephalopathy was first described in 1951 by Denis Archibald Leigh in a 7-month-old boy who developed progressive neurological deterioration triggered by infection. Autopsy revealed bilateral symmetric subacute necrotic lesions extending from the thalamus to the pons, inferior olivary nuclei, and posterior columns of the spinal cord [2]. Owing to similarities with Wernicke’s encephalopathy, abnormalities of thiamine metabolism were initially suspected [3]. In 1977, a direct association between respiratory chain dysfunction and subacute necrotizing encephalopathy was reported for the first time, establishing mitochondrial dysfunction as the underlying etiology of Leigh encephalopathy [4]. Leigh encephalopathy typically presents in early infancy with progressive psychomotor regression and symmetric necrotizing degeneration of the brainstem and basal ganglia. Diagnosis is confirmed by demonstrating mitochondrial dysfunction through biochemical assays, pathological evaluation, and genetic testing, in addition to clinical, radiological, and laboratory findings. Conditions showing similar MRI findings include neonatal asphyxia, neonatal jaundice, Wernicke encephalopathy, carbon monoxide poisoning and cyanide poisoning, organic acidurias (methylmalonic acidemia, propionic acidemia, and glutaric acidemia type 1), biotin-responsive basal ganglia disease, and storage disorders such as Wilson disease [5].

The present case describes a patient with homocystinuria due to MTHFR deficiency, an inherited metabolic disorder that is not a primary mitochondrial disease, who presented with a Leigh-like phenotype. In recent years, the concept of Leigh syndrome spectrum has been proposed, and it has become increasingly recognized that characteristic Leigh-like phenotypes can also occur in inherited metabolic disorders other than primary mitochondrial diseases [6]. Therefore, when Leigh syndrome spectrum is suspected, a comprehensive metabolic evaluation considering a broad range of inherited metabolic disorders is important.

## 2. Case Presentation

The patient was a 9-year-old girl born at term without perinatal complications. Feeding difficulties were noted from 2 months of age, and microcephaly and hypotonia were identified at a 4-month health check. She was admitted at 5 months for diagnostic evaluation. At admission, both plasma and cerebrospinal fluid lactate-to-pyruvate ratios exceeded 20, suggesting mitochondrial dysfunction. Brain MRI demonstrated symmetric T2-weighted hyperintensities in the bilateral caudate nuclei and putamina, and MR spectroscopy revealed a lactate peak in the same regions. These findings raised suspicion for Leigh encephalopathy (Figure S1).

Whole mitochondrial DNA sequencing using skeletal muscle at 9 months of age did not identify any pathogenic variants. Skeletal muscle biopsy revealed no findings suggestive of neuromuscular disease. At 5 years of age, blood-based mitochondrial DNA analysis detected a homoplasmic single-base insertion at position 15,939 in the threonine tRNA gene (NC_012920.1:m.15939_15940insT); however, because the variant had not been previously reported as pathogenic, its clinical significance remained uncertain.

At 7 years of age, the patient developed vomiting, oliguria, and somnolence during acute SARS-CoV-2 infection and was hospitalized. Regarding her functional status, she was bedridden and had no meaningful speech. Her cognitive function was severely impaired, and she was unable to communicate with others. Acute exacerbation of Leigh encephalopathy was suspected, and treatment included correction of acidosis and intensification of vitamin cocktail therapy. Although the lactate-to-pyruvate ratio on admission was <20, brain MRI revealed T2-weighted hyperintensities in the bilateral basal ganglia and thalami, with predominant peripheral hyperintensity and presumed swelling in the left basal ganglia and thalamus (Figure S2). Follow-up brain CT and MRI performed 2 weeks after admission showed ventricular enlargement and a small intraventricular hemorrhage. Progressive T2-weighted hyperintensities were seen in the globus pallidus, posterior limb of the internal capsule, putamen, and thalamus, predominantly on the right side (Figure S3). Because these findings were atypical for Leigh encephalopathy, an underlying metabolic disorder was suspected.

Metabolic screening revealed decreased plasma methionine levels and elevated homocystine in both plasma and urine. Total homocysteine levels were markedly increased (Table S1). These findings suggested homocystinuria due to MTHFR deficiency. Trio-based genomic analysis identified two rare variants in MTHFR: a paternally inherited synonymous variant (NM_005957.5(MTHFR):c.966C>T (p.Thr322=); rs138748774) classified as likely benign in ClinVar, and a maternally inherited splice-site variant (NM_005957.5(MTHFR):c.476-1G>A (rs2100562061)) classified as likely pathogenic. Measurement of MTHFR enzyme activity revealed a profound reduction across all specimens tested, including fibroblasts, lymphocytes, lymphoblasts, and dried blood spots (Table S2), confirming the diagnosis of homocystinuria due to MTHFR deficiency. Homocystinuria due to MTHFR deficiency is inherited in an autosomal recessive manner. In the present case, NM_005957.5(MTHFR):c.476-1G>A (likely pathogenic) and NM_005957.5(MTHFR):c.966C>T (p.Thr322=) (likely benign) were inherited from the mother and father, respectively, and biochemical analysis demonstrated reduced MTHFR enzyme activity. Considering these genetic and biochemical findings together, we considered that NM_005957.5(MTHFR):c.966C>T (p.Thr322=) may have contributed to the disease phenotype in this patient. Furthermore, review of the newborn screening results revealed a low methionine level of 7.22 μM (−2.58 SD).

Treatment with betaine, a methyl-group donor used for homocystinuria, was initiated. Plasma total homocysteine concentrations decreased after treatment (Figure S4), and the patient demonstrated clinical improvement, including increased alertness and improved mood. One year after initiation of betaine, brain MRI showed reduced ventricular enlargement and disappearance of the previously observed T2-weighted hyperintensities in the basal ganglia (Figure S5). Informed consent was obtained from the patient’s guardian for the publication of the patient’s clinical data.

## 3. Discussion

The term “Leigh syndrome spectrum” has recently been used as an overarching designation encompassing both classical Leigh syndrome and Leigh-like conditions, emphasizing their continuum of clinical presentations [6]. The spectrum includes atypical cases that fall within the diagnostic boundaries of Leigh syndrome but exhibit non-classical phenotypes. In the diagnostic algorithm proposed by Manuela Schubert Baldo et al., metabolic testing—including plasma amino acid profiling, acylcarnitine analysis, and urinary organic acid analysis—is considered essential for screening inborn metabolic disorders and identifying treatable etiologies [7].

The present case represents homocystinuria due to MTHFR deficiency manifesting with a Leigh-like phenotype, fitting within the Leigh syndrome spectrum. ECHS1 and HIBCH deficiencies, known disorders of valine metabolism, are established causes of Leigh syndrome spectrum [6]. Accumulation of methacrylyl-CoA or acrylyl-CoA in these disorders leads to secondary mitochondrial dysfunction. Among 19 patients in a reported cohort (13 with ECHS1 deficiency and 6 with HIBCH deficiency), 17 exhibited Leigh encephalopathy and 18 had basal ganglia lesions [8]. Although MTHFR deficiency and ECHS1/HIBCH deficiencies are not primary mitochondrial diseases, they may share a common pathophysiological mechanism in which secondary mitochondrial dysfunction resulting from metabolic disturbances contributes to the development of a Leigh-like phenotype.

Averdunk et al. described a pediatric case of classical homocystinuria presenting with acute dystonic episodes suggestive of mitochondrial disease and symmetric basal ganglia involvement [9]. Experimental in vivo studies have demonstrated that elevated homocysteine induces oxidative stress and mitochondrial damage in neuronal cells [10]. Additionally, a hypothesis has been proposed that DNA hypomethylation caused by MTHFR deficiency disrupts the epigenetic regulation of gene expression, leading to downregulation of nuclear genes involved in the electron transport chain [11]. Taken together, these findings support the concept that homocystinuria may secondarily impair mitochondrial function and manifest with a Leigh-like phenotype. In the present case, retrospective review of the newborn screening results suggests that, from the neonatal period until the diagnosis of MTHFR deficiency at 7 years of age, persistent metabolic abnormalities characterized by hypomethioninemia and hyperhomocysteinemia likely contributed to the development of central nervous system involvement.

In contrast to classical CBS-related homocystinuria, which is characterized by ectopia lentis, Marfanoid skeletal abnormalities, thromboembolism, and neurological manifestations, MTHFR deficiency predominantly presents with neurological abnormalities, including developmental delay, hypotonia, seizures, and progressive neurological deterioration. The predominance of neurological manifestations in MTHFR deficiency may be attributable not only to homocysteine accumulation but also to impaired remethylation of homocysteine to methionine, resulting in reduced availability of methionine and S-adenosylmethionine and subsequent impairment of methylation-dependent processes in the central nervous system [12]. Biochemically, CBS deficiency is associated with elevated methionine levels, whereas MTHFR deficiency typically shows low or normal methionine levels despite markedly elevated total homocysteine.

The methionine concentration of 7.22 μM was identified as being markedly low only retrospectively, after the diagnosis of MTHFR deficiency. Importantly, even if the currently proposed low-methionine screening cutoff for detecting remethylation disorders (<7.0 μM) were retrospectively applied, the patient’s methionine concentration of 7.22 μM would have been just above the cutoff and the screening result would still have been considered negative. Thus, this case illustrates an important limitation of methionine-based NBS: patients with MTHFR deficiency may be missed not only by conventional NBS programs targeting elevated methionine but also by low-methionine screening when methionine concentrations are borderline. When the patient was hospitalized at 5 months of age, Leigh encephalopathy was strongly suspected. At that time, the concept of Leigh syndrome spectrum had not yet been clearly established, and it was not widely recognized that inherited metabolic disorders other than primary mitochondrial diseases could present with a Leigh-like phenotype. Therefore, a more comprehensive metabolic evaluation was not performed. Because Leigh syndrome spectrum can be caused by potentially treatable inherited metabolic disorders, comprehensive metabolic screening should be considered in patients with Leigh syndrome spectrum.

## 4. Conclusions

Leigh encephalopathy encompasses a broad range of etiologies, and the concept of the Leigh syndrome spectrum highlights its clinical and pathological diversity. Because treatable metabolic disorders such as homocystinuria may present with a Leigh-like phenotype, comprehensive metabolic evaluation should be considered when Leigh encephalopathy is suspected.

## Data Availability

The datasets used and analyzed in this study are available from the corresponding author upon reasonable request, subject to patient privacy and confidentiality requirements.

## Supplementary Materials

The following supporting information can be downloaded at: https://www.mdpi.com/article/10.3390/children13101332/s1, Figure S1: Axial brain MRI images at 5 months of age. (A) T2-weighted brain MRI showed symmetric hyperintensities in the bilateral caudate nuclei and putamina. (B) MR spectroscopy of the symmetric T2-weighted hyperintense regions revealed a lactate peak. Figure S2: Axial brain MRI images at admission at 7 years of age. T2-weighted brain MRI showed hyperintensities in the bilateral basal ganglia and thalami. Figure S3: Axial brain CT and MRI images 2 weeks after admission. (A) Brain CT showed a small amount of hemorrhage in the right lateral ventricle. (B) Brain MRI showed ventricular enlargement and T2-weighted hyperintensities in the globus pallidus, posterior limb of the internal capsule, putamen, and parts of the thalamus, predominantly on the right side. Figure S4: Laboratory course after initiation of betaine. After initiation of betaine therapy, plasma total homocysteine levels decreased, while methionine levels increased. Figure S5: Axial brain MRI images 1 year after initiation of betaine therapy. Brain MRI showed reduced ventricular and sulcal enlargement, and resolution of the T2-weighted hyperintense lesions in the basal ganglia. Table S1: Results of metabolic screening. Table S2: Results of MTHFR enzyme activity.
