# Supplementary material for: A Pediatric Case of Homocystinuria Due to MTHFR Deficiency with Early Clinical Features Resembling Leigh Encephalopathy
Source: Children (Basel). 2026 Sep 30;13(10):1332. doi: 10.3390/children13101332 (PMC13703112; doi:10.3390/children13101332)
Supplement: Supplementary file 1 [file children-13-01332-s001.zip › children-4559946-supplementary.pdf]

Figure S1

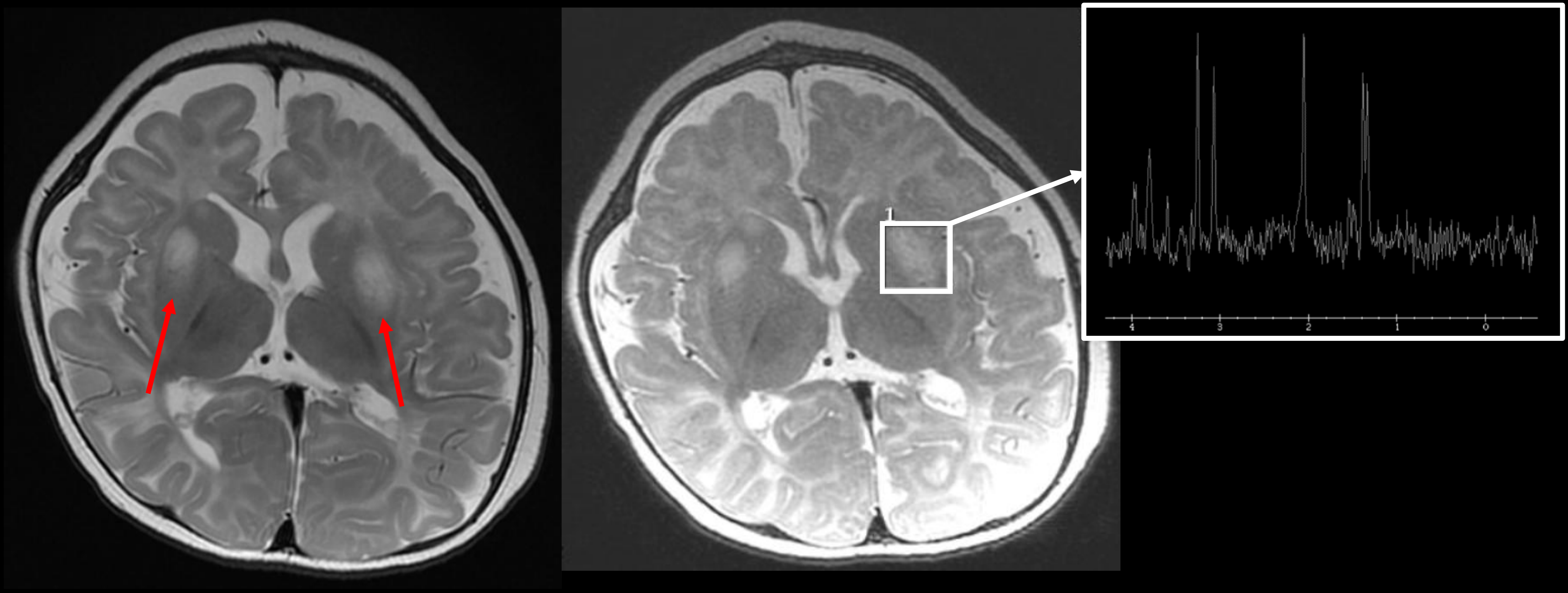

(A) (B)

Figure S2

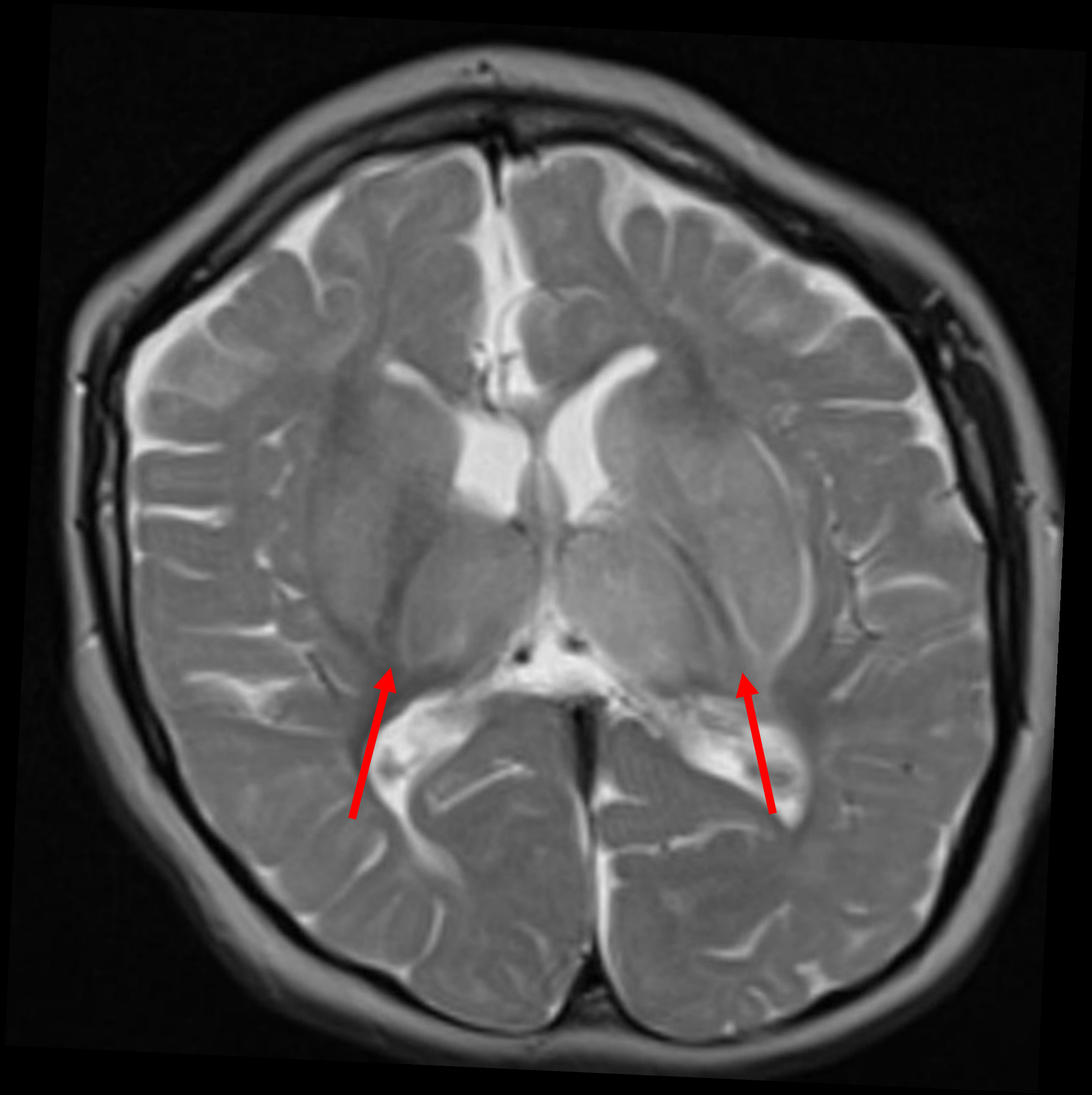

Figure S3

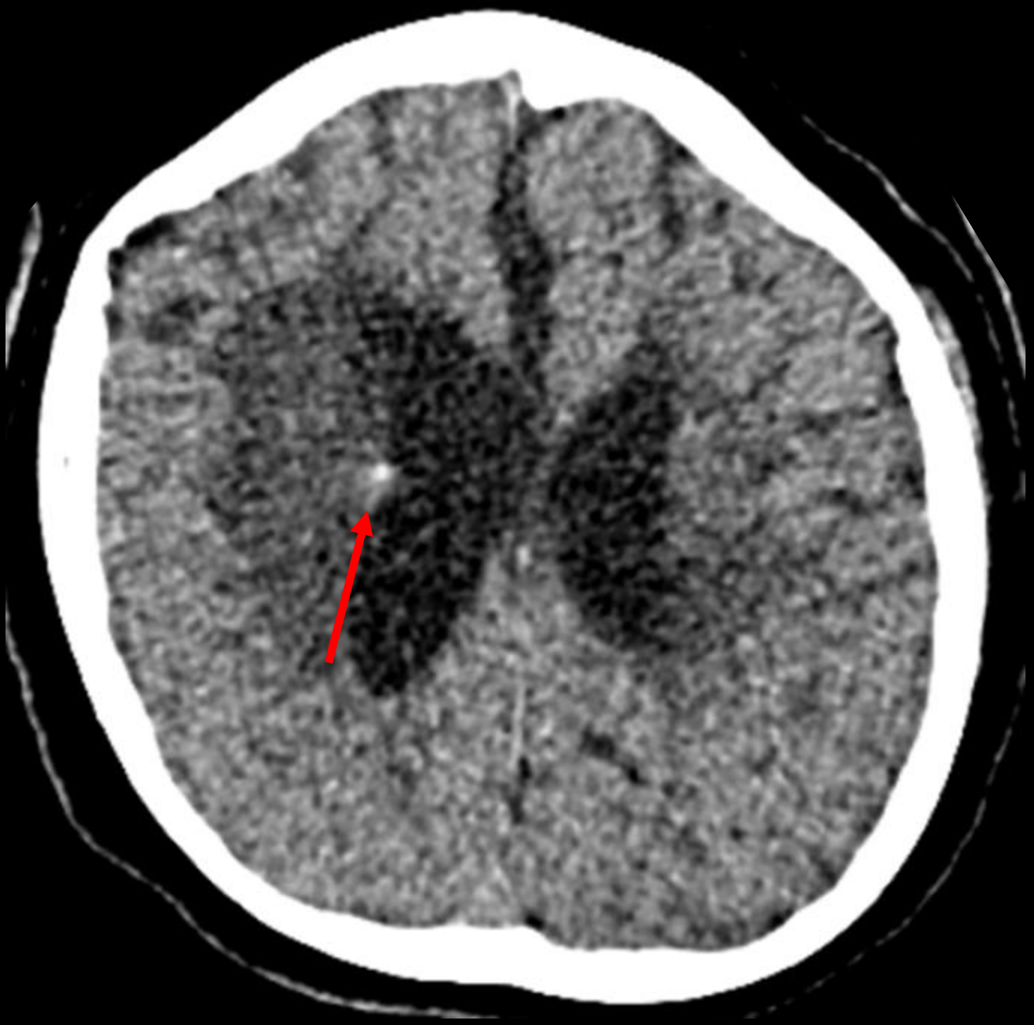

(A)

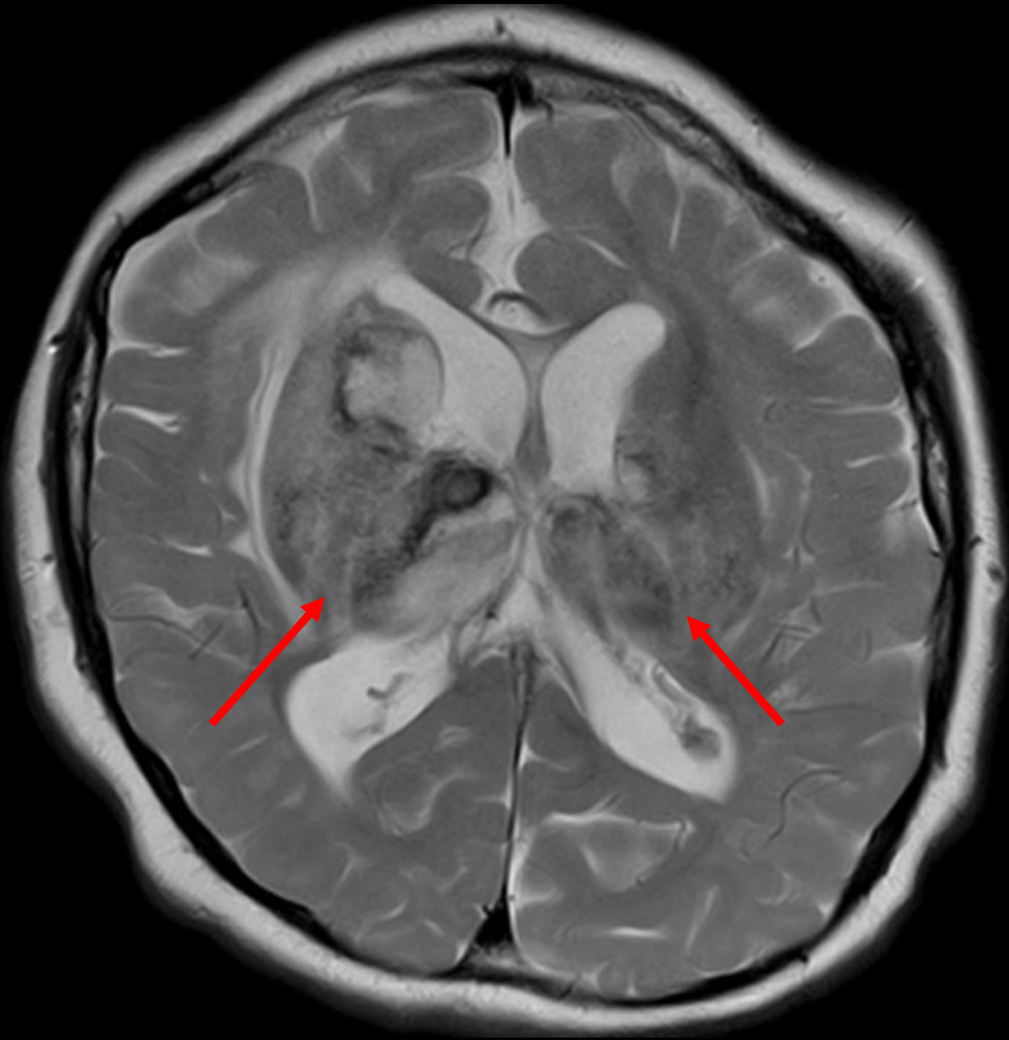

(B)

Figure S4

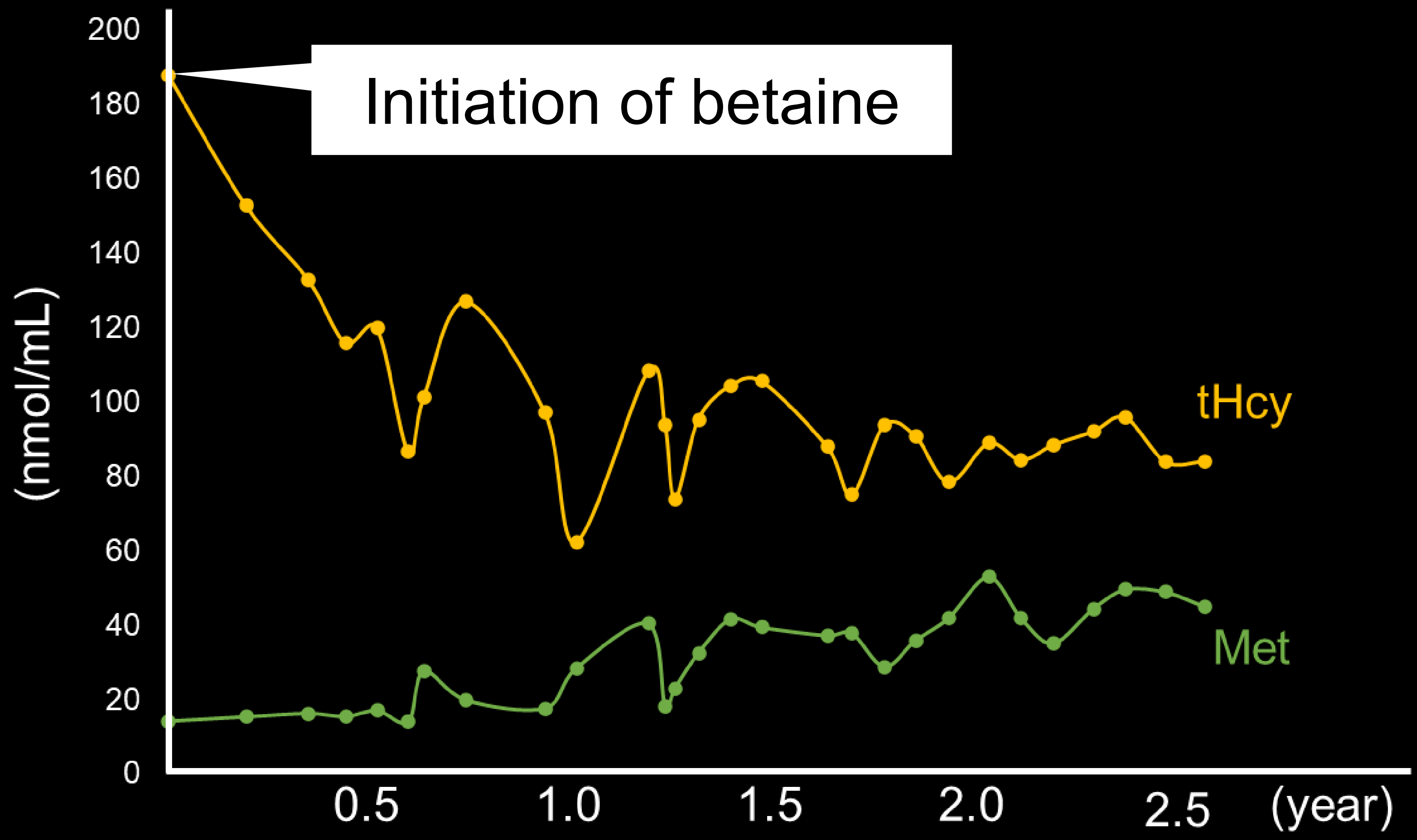
Initiation of betaine
200
180
160
140
120
100
80
60
40
20
0
(nmol/mL)
tHcy
Met
0.5
1.0
1.5
2.0
2.5
(year)

Figure S5

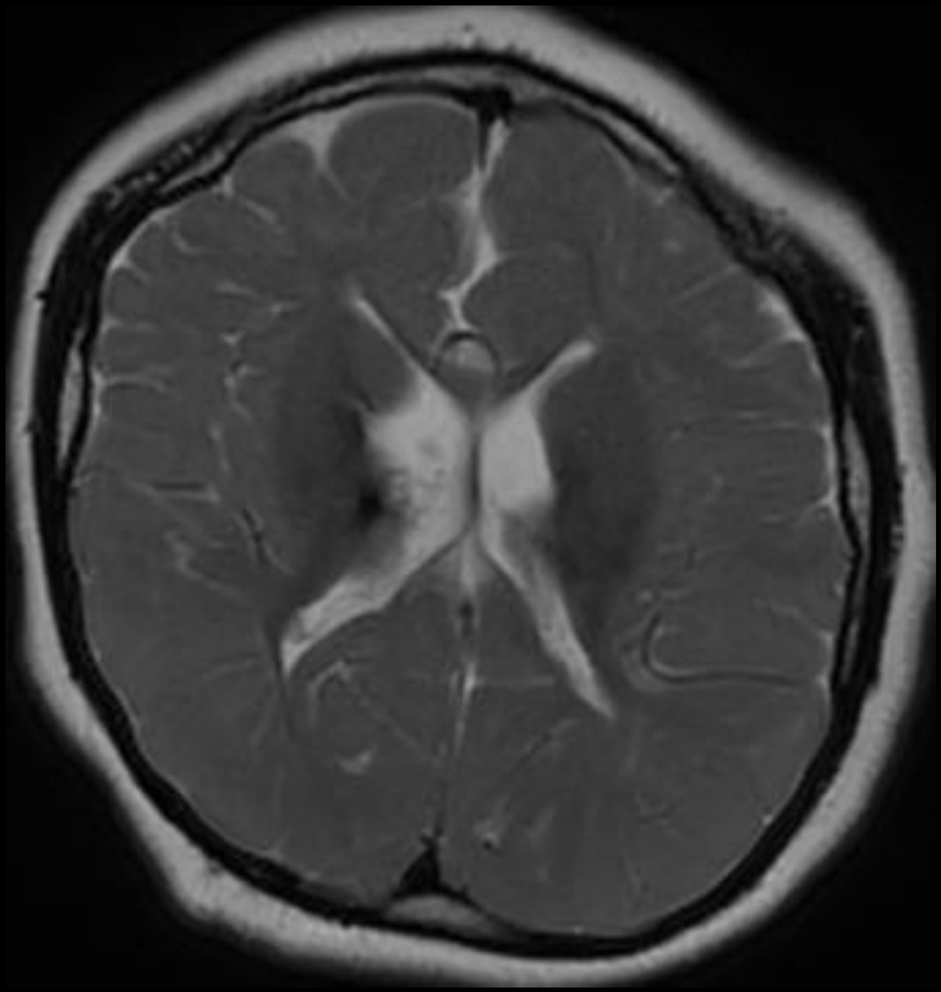

Table S1

【Metabolic screening】

	Blood(nmol/mL)	Urine（μmol/g·Cre）
Methionine(Met)	14.0(18.9-40.5)	12.2(TR-20.2)
Homocystine(Homocys)	23.3(ND)	237.9(ND)

ND: below the limit of detection
TR: trace amount

	Blood(nmol/mL)	Urine（μmol/L）
Total Homocysteine(tHcy)	187.8(>60)	5,968(markedly elevated)

Table S2

Fibroblast extracts	MTHFR activity (µU/mg protein)	MTHFR activity (% of mean control value)
Patient(MTHFR deficiency Patient)	2.96(2.99-44.0)	0.92 %
Controls value mean(range)	320.8(240.1-393.9)	

Lymphocyte extracts	MTHFR activity (µU/mg protein)	MTHFR activity (% of mean control value)
Patient(MTHFR deficiency Patient)	7.12(3.71-7.25)	1.06 %
Controls value mean(range)	669.8(225.8-1161.8)	

Lymphoblast extracts	MTHFR activity (µU/mg protein)	MTHFR activity (% of mean control value)
Patient(MTHFR deficiency Patient)	6.23(5.44-20.0)	1.64 %
Controls value mean(range)	380.5(147.0-699.1)	

Dried blood spot extracts	MTHFR activity (µU/mg protein)	MTHFR activity (% of mean control value)
Patient(MTHFR deficiency Patient)	8.08(0.0-12.1)	1.88 %
Controls value mean(range)	429.9(25.4-872.3)	
